# Extracorporeal Photopheresis in Tandem With Continuous Renal Replacement Therapy: A Case Report

**DOI:** 10.1002/jca.70170

**Published:** 2026-08-18

**Authors:** M. S. Reddy, J. Bardahl, A. Nord, T. Hardy

**Affiliations:** ^1^ Department of Pathology and Laboratory Medicine Indiana University School of Medicine Indianapolis Indiana USA; ^2^ Pediatric Hematology Oncology and Stem Cell Transplant, Department of Pediatrics Indiana University School of Medicine Indianapolis Indiana USA; ^3^ Riley Hospital for Children at Indiana University Health Indianapolis Indiana USA; ^4^ Indiana University Health Physicians Indianapolis Indiana USA

## Abstract

The use of therapeutic plasma exchange in tandem with continuous renal replacement therapy (CRRT) and extracorporeal membrane oxygenation (ECMO) has been well described; however, evidence showing the use of extracorporeal photopheresis (ECP) in tandem is lacking. Here we report a consult to perform ECP in tandem with CRRT for a 26‐year‐old female with a history of severe aplastic anemia who underwent allogeneic hematopoietic stem cell transplant, complicated by steroid‐refractory acute graft‐versus‐host disease, vaso‐occlusive disease, thrombotic microangiopathy, adenoviremia, and acute renal and respiratory failure. We performed six tandem ECP procedures (seven total) over 19 days, until the patient's decline resulted in compassionate discontinuation of care. During our procedures, CRRT managed the ionized calcium and adjusted the calcium infusion rate accordingly. We encountered red blood cell and optic bowl sensor alarms during ECP. This case, to our knowledge, is the first to describe the inpatient use of ECP in tandem with CRRT.

## 1 Introduction

Tandem access into an existing continuous renal replacement therapy (CRRT) and/or extracorporeal membrane oxygenation (ECMO) circuit has been utilized to allow concurrent therapeutic plasma exchange in critically ill patients [1, 2, 3]. While not previously described, extracorporeal photopheresis (ECP) may also conceivably be performed in tandem with CRRT and/or ECMO. Key factors to consider include the circuit configuration (parallel versus series), blood flow rates, extracorporeal volume, management of electrolytes, and adverse reactions. Here we describe our experience with our first consult to perform inpatient ECP in tandem with CRRT. This is a single patient case report and is not considered human subjects research; IRB approval was not considered necessary. The patient's legal representative signed an informed consent, authorizing the release of this information for publication.

## 2 Case Report

The patient was a 26‐year‐old female who underwent allogeneic hematopoietic stem cell transplant (HSCT) for severe aplastic anemia complicated by multisystem organ failure, vaso‐occlusive disease, and transplant‐associated thrombotic microangiopathy, requiring intubation in the setting of septic shock. She was found to have skin manifestations and worsening acute graft‐versus‐host‐disease (aGVHD). She was treated with intravenous methylprednisolone with minimal improvement, prompting an urgent consult for ECP due to concerns for steroid‐refractory aGVHD [4, 5, 6]. The American Society for Apheresis considers ECP for aGVHD a category II indication, with grade 1B evidence [5].

We initiated ECP on day 42 post‐HSCT using the Therakos CELLEX Photopheresis System (CELLEX main software 05.18 [CRC:0670]); Therakos Inc. USA. CRRT was performed using the TrimedX system and a Prismaflex HF1000 filter, second generation (Baxter International Incorporated, Illinois—USA). Procedures were conducted twice weekly, following institutional protocol, via central venous access using double‐needle mode. Our draw and return lines were configured in a parallel circuit and were connected to the CRRT return line (see Figure S1). Anticoagulant Citrate Dextrose A was used as the anticoagulant at a standard anticoagulant‐to‐whole blood ratio of 1:10 to 1:8. Calcium chloride was used as supplementation, and the ionized calcium (iCal) target was 1.10–1.30 mmol/L and checked every 15 min, managed by the CRRT nurse. The patient's total blood volume (TBV) was 4172 mL and the CRRT circuit volume was 165 mL; thus, a total of 4337 mL was used for apheresis TBV calculations. Prior to each procedure, the patient's hematocrit was > 27%. Table 1 contains the mean tandem ECP and CRRT procedure parameters.

**TABLE 1:** Mean values for tandem ECP and CRRT procedure parameters (6 tandem procedures).

Category	Parameter	Mean (range)	Units
ECP—Flow rates	Initial collect rate	38.33 (30–50)	mL/min
	Ending collect rate	43.33 (30–50)	mL/min
	Initial return rate	43.33 (35–55)	mL/min
	Ending return rate	48.33 (35–55)	mL/min
ECP—Volumes	Whole blood processed	1527.17 (1328–1592)	mL
	Treatment volume	160.33 (133–224)	mL
	Instrument AC volume	214.17 (200–228)	mL
	AC volume to patient	184.17 (170–198)	mL
	Apheresis saline volume	20.83 (10–40)	mL
ECP—Medication & processing	UVADEX dose	2.68 (2.20–3.80)	mcg
	UVA photo time	34.45 (30.11–43.25)	min
ECP—Fluid balance	Ending fluid balance	12.33 (9–16)	mL
	CELLEX fluid balance	367.67 (146–420)	mL
	Final apheresis fluid balance	430.17 (415–449)	mL
CRRT—Flow settings	Blood flow rate	180 (180–180)	mL/min
	Dialysate flow rate	1000 (1000–1000)	mL/hr
	Replacement fluid rate	900 (900–900)	mL/hr
	Total clearance	1900 (1900–1900)	mL/hr
CRRT—Fluid management	Total fluid removed	792.78 (474.27–1449.10)	mL
CRRT—Citrate & calcium	Calcium/citrate ratio	9.04 (8.8–9.6)	—
	Calcium chloride infusion	46.36 (39–75)	mL/hr
	Pre‐iCal	0.94 (0.52–1.24)	mmol/L
	Post‐iCal	1.11 (1.05–1.18)	mmol/L

Abbreviations: AC = anticoagulant; CRRT = Continuous renal replacement therapy; ECP = Extracorporeal photopheresis; hr = hour; iCal = Ionized calcium; L = liter; mcg = microgram; min = minutes; mL = milliliter; mmol = millimole; UVA = Ultraviolet A; UVADEX = methoxsalen, 8‐methoxypsoralen.

We performed six ECP procedures in tandem with CRRT. The average procedure length was 102 min. Red blood cell and optic bowl sensor alarms were the ECP complications encountered, as the sensor had difficulty identifying an optimal interface. Our default optic bowl sensor is 150 and on the 5th procedure it was adjusted to 200. To complete the last and 7th procedure (not performed in tandem), we stopped and restarted the procedure twice and changed the optic bowl sensor to 90. Difficulty identifying the appropriate interface was likely caused by the patient's underlying thrombotic microangiopathy. We did not experience any pressure alarms, despite the difference in ECP and CRRT flow rates. The CRRT team managed iCal levels and remained within an average range of 0.94–1.11 mmol/L across procedures. This is lower than the targeted iCal range, however, because the patient did not experience hypocalcemia related side effects, we maintained our standard citrate use. Despite ECP, the patient's status declined from fungemia and viremia until compassionate extubation and removal from CRRT was pursued.

## 3 Discussion

This case provides the evidence to perform ECP procedures in tandem with other extracorporeal treatment modalities, in this case CRRT. We recommend a parallel circuit configuration due to the large difference in flow rates between ECP and CRRT and would recommend this same configuration for other circuits. Nurses performing tandem ECP procedures should have experience troubleshooting alarms and adjusting the optic bowl sensor, as this was necessary for our procedures. One well‐known concern in patients undergoing apheresis in tandem with other modalities is potential calcium instability [3, 7]. We encountered low iCal values at the start of our procedures which were quickly increased after starting. Despite low pre‐iCal values, the patient did not experience hypocalcemia related side effects. We recommend starting with baseline ECP and CRRT citrate and calcium and adjusting based on the patient's clinical response. For future tandem cases, we will likely utilize an ECP anticoagulant‐to‐whole blood ratio > 1:10 to maintain higher iCal levels. This case demonstrates the safety and feasibility of performing ECP in tandem with other circuits, primarily CRRT.

## Funding

The authors have nothing to report.

## Conflicts of Interest

Andrew Nord, PA‐C—American Society for Apheresis (ASFA) board, secretary.

## Supporting information


**Figure S1:** ECP and CRRT tandem venous access connections; circuits connected in a parallel configuration. ECP = Extracorporeal photopheresis; CRRT = Continuous renal replacement therapy.

## Data Availability

The data that support the findings of this study are available on request from the corresponding author. The data are not publicly available due to privacy or ethical restrictions.
