# Supplementary material for: Extracorporeal Photopheresis in Tandem With Continuous Renal Replacement Therapy: A Case Report
Source: J Clin Apher. 2026 Aug 18;41(4):e70170. doi: 10.1002/jca.70170 (PMC13485702; doi:10.1002/jca.70170)
Supplement: Supplementary file 1 [file JCA-41-e70170-s001.docx]

**Supplementary Figure 1:** ECP and CRRT tandem venous access connections; circuits connected in a parallel configuration

ECP = Extracorporeal photopheresis; CRRT = Continuous renal replacement therapy
